# Optimization of candidate-gene SNP-genotyping by flexible oligonucleotide microarrays; analyzing variations in immune regulator genes of hay-fever samples

**DOI:** 10.1186/1471-2164-8-282

**Published:** 2007-08-17

**Authors:** Janne Pullat, Robert Fleischer, Nikolaus Becker, Markus Beier, Andres Metspalu, Jörg D Hoheisel

**Affiliations:** 1 Division of Functional Genome Analysis, Deutsches Krebsforschungszentrum, Im Neuenheimer Feld 580, 69120 Heidelberg, Germany; 2 Division of Clinical Epidemiology, Deutsches Krebsforschungszentrum, Im Neuenheimer Feld 580, 69120 Heidelberg, Germany; 3 Institute of Molecular and Cell Biology, 23 Riia St., 51010 Tartu, Estonia; 4 Febit biotech, Im Neuenheimer Feld 517, 69120 Heidelberg, Germany; 5 The Estonian Biocentre, 23b Riia St., 51010 Tartu, Estonia; 6 Molecular Diagnostics Centre, United Laboratories of the Tartu University Hospital, 1a Puusepa Str., 50406 Tartu, Estonia; 7 Estonian Genome Project of University of Tartu, 61b Tiigi Str., 50410 Tartu, Estonia

## Abstract

**Background:**

Genetic variants in immune regulator genes have been associated with numerous diseases, including allergies and cancer. Increasing evidence suggests a substantially elevated disease risk in individuals who carry a combination of disease-relevant single nucleotide polymorphisms (SNPs). For the genotyping of immune regulator genes, such as cytokines, chemokines and transcription factors, an oligonucleotide microarray for the analysis of 99 relevant SNPs was established. Since the microarray design was based on a platform that permits flexible *in situ *oligonucleotide synthesis, a set of optimally performing probes could be defined by a selection approach that combined computational and experimental aspects.

**Results:**

While the *in silico *process eliminated 9% of the initial probe set, which had been picked purely on the basis of potential association with disease, the subsequent experimental validation excluded more than twice as many. The performance of the optimized microarray was demonstrated in a pilot study. The genotypes of 19 hay-fever patients (aged 40–44) with high IgE levels against inhalant antigens were compared to the results obtained with 19 age- and sex-matched controls. For several variants, allele-frequency differences of more than 10% were identified.

**Conclusion:**

Based on the ability to improve empirically a chip design, the application of candidate-SNP typing represents a viable approach in the context of molecular epidemiological studies.

## Background

Array-based technologies are revolutionizing genomics, especially the analysis of DNA variation. Array technologies are not without limitations, however, and one major drawback is the poor flexibility of typical array formats. It is cumbersome to create one's own tailored arrays by spotting DNA. Commercially available microarrays, on the other hand, either contain a fixed and usually broadly applicable content or are expensive to purchase with customized features. The fixed-content arrays are useful for taking advantage of the high resolution genetic map of the human genome that is based on single nucleotide polymorphisms [1,1], which define DNA blocks (haplotypes) [1]. Since SNPs are the most common type of genetic variation between individuals, it makes sense to utilize them for the localization of disease genes by identifying haplotypes that are associated with phenotypic traits, especially in the case of multifactorial diseases [1-5]. As a consequence of such a study, however, further analysis is required for improving the resolution of the mapping process or trying to identify the polymorphisms that are actually responsible for the phenotypic variation. Alternatively to the process described above, one can immediately focus on the analysis of particular polymorphisms in candidate genes, if circumstantial evidence indicates their possible relevance to the occurrence of a disease. In either approach the production of a customized microarray is required. Also, experience has demonstrated the need for a careful design of the experimental set-up in order to avoid unacceptable error [6].

Irrespective of the algorithm used for the sequence selection of the probe set, the final functional test of the suitability of an oligonucleotide array for genotyping results from an empirical analysis of the hybridization performance of the oligonucleotide probes. In consequence, it is likely that the initial chip design will be changed by replacing ill-performing oligonucleotides with alternative sequences. For this process, the ability to easily change the chip layout is essential. Light-induced *in situ *synthesis controlled by a micro-mirror device [7,8] combines high synthesis yields of more than 99.5% per condensation [9] – and therefore good oligonucleotide quality – with the power of producing oligomer arrays of high density, reproducible characteristics and flexible layout.

In this study, we present the process of establishing an oligonucleotide microarray based on an on-site *in situ *synthesis technology for typing DNA samples in immune regulator genes including cytokines, chemokines and transcription factors. Genetic variants in immune regulator genes have been associated with numerous diseases, including allergies and cancer, with apparently an elevated disease risks in individuals that carry a combination of disease-relevant SNPs. For the array design, we exploited the flexibility of the GeniomOne device [8]. It employs a digital projector to synthesize oligonucleotide array features within channels of a three-dimensional micro-fluidic reaction carrier. The system allows the synthesis of a probe set of up to 64,000 oligonucleotides on a single chip, which subsequently can be hybridized with up to eight samples. For this analysis a microarray that assays 99 relevant SNPs was established by an iterative cycle of probe design and experimental evaluation. Subsequently, the performance of this microarray was investigated in a pilot study. Hay-fever patients aged 40–44 that exhibited high IgE levels against inhalant antigens and an age and sex-matched control group were analyzed.

## Results

From a case-control study on hay-fever [10], 19 cases with the most extreme plasma IgE levels against inhalant antigens and 19 age- and sex-matched non-atopic controls were selected for the project. Originally, 141 SNPs in cytokine genes and other immune regulatory factors were selected from published studies and SNP-databases [11-13]. If possible, SNPs with known or potential functional relevance and allele frequency information were selected. Also, sequence complexity between the probes was meant to be similar, since it is well established that the rate of reassociation depends on sequence complexity [14]. In addition, the initial compilation was based on theoretical calculations of interactions between all oligonucleotide probes and PCR fragments. The program "SNP CrossChecker" by Febit GmbH was used to check the cross reactivity between oligoprobes and template sequences reducing the number of PCR-products by 13 to 128. The threshold of maximally possible homology between 23 mere oligoprobe and template sequences was set to 85%. It takes into account that if within the 23 nucleotides of a probe, 20 nucleotides will basepairing with a template, this will produce sufficiently stable complex to produce false positive signals in the genotyping analysis.

**Figure 1:**
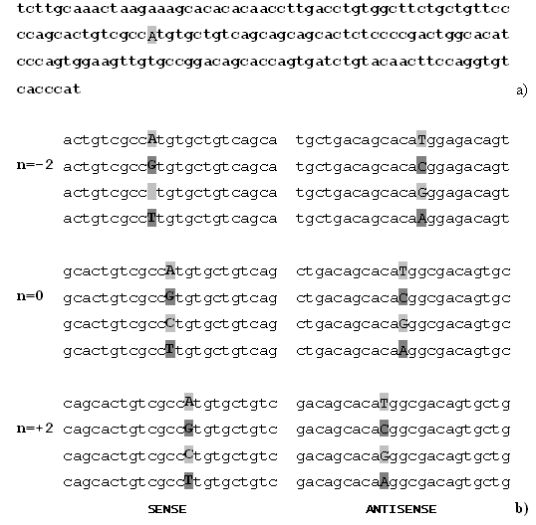
**Design a 23 mer oligonucleotide for SNP detection**. In (a) the relevant PCR-product of 166 bp is shown. (b) exhibits the set of oligonucleotides (12 for sense and 12 for antisense strand; at n = 0 the allele is located in the middle of the oligomer, at n = -2 and n = +2 the SNP is shifted by 2 nucleotides to the left and right, respectively.

Theoretically the probe properties could be assessed basis their sequence similarity and hybridization properties. Experimentally "bad" probe has low specificity, sensitivity and uniformity under given reaction conditions (temperature, base composition, salt concentration, hybridization time). Specificity and stability of DNA duplex formation strongly depend on sequence and base composition [15,16]. Also, the target sequence on either side of the SNP position plays an important role since secondary structures may strongly affect the hybridization behavior of a sample [17]. Therefore, it is frequently insufficient to predict hybridization performance merely on the basis of theoretical calculations. Consequently, we analyzed and optimized the experimental parameters of SNP position in the oligonucleotide and the overall length of the probes as well as hybridization temperature and duration. For each SNP, all four possible sequence variations were applied to the chip. One of the probes is designed to be perfectly complementary to a short stretch of the reference sequence (perfect match – PM) and the other three are identical to the first, except at the interrogation position, where one of the other three bases is substituted (mismatches – MM). PM/MM scheme enables in addition subtract directly both the background level and cross-hybridization signals providing thus with redundancy required for the reliable microarray analysis. The perfect match probe (PM) is designed complementary to the target sequence and the so-called mismatch probe (MM) is identical with the PM, except the base in the middle of the sequence. Ideally, there is 30-fold difference in the signal intensities of PM vs. MM oligo. In hybridization the oligo signal intensity depends directly of its sequence GC content. Depending on sequence content (high G/C content) the MM oligo can result sufficiently high signal and interfere discrimination between PM and MM signals. In such cases the entire set of 24 oligoprimers, specially designed for detection of one SNP from sense and antisense strands, is underperforming and has to be left out of array design. In addition, we tested positional effects by moving the polymorphic nucleotide from the center to positions +2 and -2 as well as +1 and -1 of the oligonucleotide probes (Fig. 1). This shift resulted in differences in signal intensities but did not add to the overall amount of information that could be gathered from an experiment. In consequence, we decided to use only probes that contained the respective SNP in a central position but placed three copies of the same oligosequence at different locations of the microarray.

Furthermore, different temperatures for hybridisation (40°C, 45°C, 50°C, and 55°C) and changes in hybridisation time from one to four hours were compared. The time of hybridisation in this experiment had little influence on number of correct and false signals. However, increased hybridisation temperature at 50°C or 55°C reduced cross hybridisation at least 5% and lowered general amount of positive signal to 60% and 40% (respectively). Reduced stringency by decreased hybridisation temperature maximized the overall number and intensity of signals, but this was accompanied with 30% increase of unspecific hybridisation signals.

**Figure 2:**
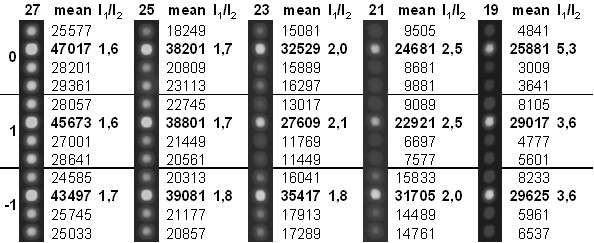
**The dependence of signal intensity on oligonucleotide length**. Hybridization was done at 45°C. *I*_1_*/I*_2 _labels the ratio of the signal at the full-match oligonucleotide and the signals at the mismatched oligonucleotides. 27, 25, 23, 21 and 19 indicates the length of oligomers. The SNP was located either at the center of the oligonucleotides (0) or shifted by two bases in either direction (+1, -1).

**Figure 3:**
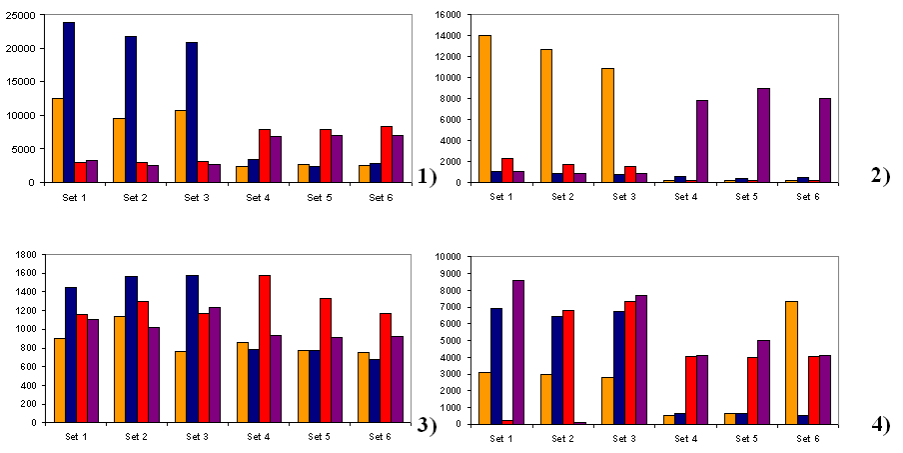
**Differences in the performance of oligonucleotides**. Set 1 to 6 label the oligonucleotides designed for detecting a SNP. Each column indicates the signal intensity at the oligomers that represent (left to right) the A, G, C or T variant of a sequence. Sets 1 to 3 are the data produced on replicate microarray positions that represent one strand, while sets 4 to 6 indicate the signal intensities produced by the complementary DNA strand. Panel (1) shows the result obtained for a heterozygous sample, panel (2) a homozygous sample. Panel (3) exhibits data obtained with an oligonucleotide that was predicted *in silico *to perform well but failed in the experiment. In panel (4) a result of an oligomer with a high degree of cross-hybridization is presented.

We also varied probe length, synthesizing on the same chip oligonucleotides of 19, 21, 23, 25 and 27 nucleotides. While longer sequences usually produce higher signal intensities, shorter oligonucleotides permit better discrimination of single base differences due to the more pronounced destabilizing effect of a mismatch. As expected, the signal intensities of both the fully matched (I_1_) and the mismatch probes (I_2_) increased with length while discrimination (I_1_/I_2_) improved the shorter the oligonucleotides were (Fig. 2). Measured signal intensity (I_1_) increases clearly with higher nucleotides number in the sequence of oligonucleotide-probe: I_1 _(27 bp) > I_1 _(19 bp) (Fig. 3). Same effect is obtained for MM (I_2_) oligo-probes as well. Though the discrimination between PM/MM according to the calculated relation of measured intensities (I_1_/I_2_) is higher for shortest set of oligo-probes as 19 bp (5,3...3,6) and the lowest with 27 bp (1,7...1,6) ones. Variation of I_1_/I_2 _among probes within the same number of nucleotides comes mainly from GC content differences/variations of probe-sequence itself. Why longer MM sequences give higher signal comparing to shorter ones comes mainly from weaker destabilizing effect of noncomplimentary nucleotide on formation of double-stranded complex between probe and target DNA.

Tests at different hybridization temperatures (40–55°C) produced the overall best results for the majority of SNPs with 23-mer probes and 3 to 4 hours of hybridization at 45°C. Finally, the selected set of oligoprobes, as well as the hybridization conditions, were tested in addition with 4 genomic DNA samples of control individuals. These control experiments had 5-fold redundancy. Concordance of analyzed genotypes were compared individually.

For selecting the best performing oligoprobes in the initial optimization experiments one test-DNA with good quality was used. All hybridization reactions from chip design step were repeated 3 times. During the optimization process, we identified several oligonucleotide probes that did not perform irrespective of the chosen hybridization conditions (e.g., Fig. 2). Apparently, the previously described selection basis of cross-reactivity could be even more stringent e.g. we should allow less base pairing. Following experimental tests revealed additional oligoprobe sets falling out from final chip design because of the same reason. Herewith, basis on experimental results, the threshold for software based oligo probe selection could be set on 80% allowing less base pairing (and less false signals due to nonspecific oligo hybridization) than 85%. In total, 29 out of the 128 SNPs (22%) could not be analyzed adequately. The respective oligomer probes that had been defined as good by the *in silico *selection process were empirically found to be ill-performing in real hybridizations. Either the absolute signal intensity was too low to permit a statistically solid analysis or the discriminative effect was insufficient. The ratio between PM and MM oligo signal intensities is supposed to be at least 1/3 (Fig. 3). The high number of failing oligonucleotides illustrates the need for a careful experimental validation of *in silico *designed microarrays.

**Figure 4:**
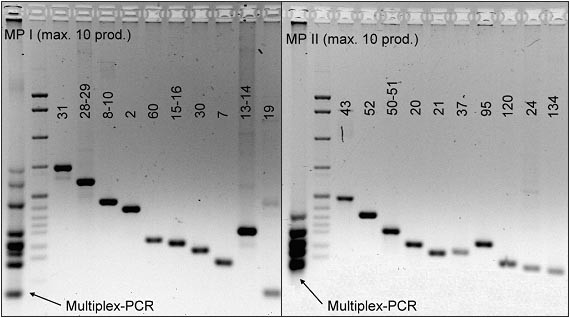
**Gel-electrophoretic separation of the products of multiplex-PCR**. Two decaplex amplifications are shown in comparison to the respective individual reactions. In both cases presented here, one product was not amplified in the multiplex reaction while the reaction worked fine in the individual amplification.

**Figure 5:**
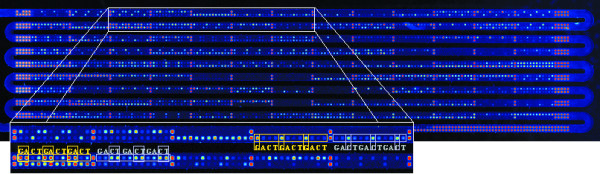
**Image of a simultaneous hybridization of 99 PCR-products to an *in situ *synthesized oligonucleotide microarray**. Usually, the features were scrambled across the array. For illustrative purposes, they were placed next to each other in this particular experiment.

Using the optimized microarray, we performed genotyping analyses at 99 SNPs in 68 genes that have a putative functional significance for the occurrence of hay-fever. From a case-control study on hay-fever [10], 19 cases with the most extreme plasma IgE levels against inhalant antigens and 19 age- and sex-matched non-atopic controls were selected. Informed consent of the participants was given in writing and the local ethics committee approved the study. PCR-amplifications of the relevant DNA-regions were performed either individually or in pools of 5 or 10 samples. While all pentaplex reactions yielded a product for each individual band, two decaplex amplifications failed to produce 1 out of the expected 10 amplicons (Fig. 4). The 99 products were pooled prior to labelling and hybridized concomitantly (Fig. 5). For each sample, analysis was repeated up to four times. The observed allele frequencies are presented in Table 1. To assess the accuracy of the genotyping, ten PCR-products of heterozygote calls obtained from the microarray analyses were subjected to gel-based DNA sequencing for confirmation. In all cases, the results were in full agreement.

**Table 1:** Relative allele frequences of SNPs genotyped in 19 hay fever patients with extreme IgE phenotype and 19 non-atopic controls.

**SNP Nr.**	**SNP name**	**SNP identifier**	**Allele 1**	**Relative frequence of allele 1 in case sample**	**Relative frequence of allele 1 in control sample**
1	IL-2_1	rs2069772	T	0.58	0.83
2	IL-2_2	rs2069763	G	0.67	0.61
3	IL-10_4	rs1800894	G	1.00	1.00
4	IL-10_5	rs1800871	C	0.77	0.83
5	IL-10_6	rs1800872	C	0.77	0.83
6	TNFA_7	rs11565	C	0.85	0.92
7	TNFA_8	rs673	A	0.03	0.03
8	TNFA_9	rs1800629	A	0.16	0.05
9	TNFA_10	rs361525	A	0.11	0.03
10	IL4_11	rs2243246	T	0.95	0.86
11	IL4_12	rs2243250	C	1.00	1.00
12	IL4_13	rs34185442	C	1.00	1.00
13	IL4_14	rs2970874	C	1.00	0.97
14	IL6_16	rs1800797	G	0.63	0.60
15	IL6_16	rs1800796	G	1.00	1.00
16	IL6_17	rs1800795	G	0.63	0.47
17	IL4R_18	rs1801275	A	0.87	0.68
18	IL4R_19	rs1805011	C	0.05	0.13
19	IL4R_20	rs8832	G	0.75	0.62
20	IL4R_21	rs1805015	T	1.00	0.89
21	IL4R_22	rs1805010	A	0.70	0.56
22	IL12p40_23	rs3124	C	1.00	1.00
23	STAT6_24	rs167769	A	0.32	0.20
24	STAT6_25	rs324015	G	0.72	0.82
25	STAT6_26	rs703817	G	0.50	0.38
26*	STAT6_27	rs4559	A	0.27	0.25
27	IFNG_28	rs2234685	A	1.00	1.00
28	IFNG_29	rs1861493	T	0.69	0.69
29	IFNG_30	rs2234687	C	1.00	1.00
30*	IFNG_31	rs2430561	T	0.50	0.38
31	IFNGR2_34	rs1802585	C	1.00	1.00
32	IFNGR2_35	rs1059293	T	0.39	0.50
33	IFNGR2_36	rs9808753	A	0.86	0.94
34	IRF1_37	rs839	G	0.74	0.89
35*	IRF1_38	rs9282762	A	0.42	0.60
36*	IRF2_40	rs1131553	G	0.32	0.50
37	IL8_41	rs1175	A	0.41	0.47
38	IL8_42	rs2227307	G	0.42	0.50
39	IL13_43	rs20541	G	0.89	0.79
40	IL13_44	rs1800925	C	0.88	0.82
41*	IL18_47	rs1946518	G	0.63	0.67
42*	IL18_48	rs1946519	C	0.87	0.71
43*	IL1B_49	rs16944	T	0.16	0.15
44	IL1B_50	rs1143627	C	0.36	0.38
45	IL1B_51	rs1799916	T	1.00	1.00
46	IL1A_52	rs17561	G	0.64	0.74
47	IL1A_53	rs1800587	T	0.36	0.31
48	IL9_56	rs1799962	A	1.00	1.00
49	TNFR1_60	rs1800692	C	0.63	0.53
50	TNFR1_61	rs1800693	A	0.53	0.72
51*	TNFRSF6_62	rs2234768	T	0.00	1.00
52	LTA_65	rs1800683	A	1.00	1.00
53	LTA_66	rs1041981	A	0.29	0.19
54	LTA_67	rs909253	G	0.25	0.19
55	IL1RN_68	rs2234676	G	0.86	0.74
56	IL1RN_69	rs419598	T	0.87	0.88
57	CTLA4_70	rs2384137	G	0.11	0.06
58	NFKBIA_72	rs1800439	G	0.53	0.56
59	IL8RB_77	rs2230054	T	0.06	0.17
60	ICAM1_78	rs1799969	A	1.00	0.97
61*	ICAM1_79	rs5498	G	0.61	0.64
62	IL3_81	rs40401	G	0.95	0.94
63	IL3_82	rs31480	G	0.84	0.82
64	MCP1_87	rs4611511	A	0.89	0.92
65	MCP1_88	rs34020694	A	0.87	0.87
66	RANTES_89	rs2107538	G	0.88	0.91
67	RANTES_90	rs2280788	C	0.95	0.95
68	CCR5_91	rs1799863	A	0.03	0.00
69	CCR2_94	rs1799865	T	0.50	0.56
70	C5_95	rs17611	G	0.66	0.50
71	C5_96	rs17612	C	0.11	0.06
72	P2X7_97	rs3751143	C	0.06	0.03
73	IL7R_106	rs1494555	G	0.34	0.35
74	PRF1_107	rs885822	T	0.83	0.94
75	TLR2_108	rs1804965	G	1.00	1.00
76	TCL1B_109	rs1064017	G	0.44	0.56
77	CCR5_110	rs1800452	G	1.00	1.00
78	IL11_111	rs1126757	A	0.38	0.44
79	IL11_112	rs2298885	G	0.61	0.85
80	IL8RA_117	rs2234671	G	0.40	0.44
81*	IL1L1_118	rs1800930	A	0.78	0.83
82*	CD36_119	rs1334512	G	0.96	0.85
83	VDR_121	rs1544410	G	0.31	0.44
84*	VDR_122	rs7975232	T	0.73	0.54
85	IL5RA_123	rs2290610	A	0.83	0.61
86	IL5R_124	rs2069812	C	0.79	0.78
87	IL5R_125	rs2069818	C	1.00	1.00
88	CX3CR1_126	rs3732379	G	0.68	0.67
89	CX3CR1_127	rs3732378	C	0.78	0.74
90*	TNFRSF1B_128	rs1061622	T	0.88	0.65
91	TNFRSF1B_129	rs1061624	A	0.31	0.47
92	TNFRSF1B_130	rs3397	T	0.88	0.79
93*	TNFRSF1A_131	rs887477	G	0.54	0.35
94	TNFRSF1A_132	rs4149570	G	0.76	0.53
95	IL4R_135	rs1805016	T	1.00	1.00
96	IL6_137	rs20069860	A	0.97	1.00
97*	IL9_138	rs20069885	C	1.00	0.96
98*	NKFB_139	rs1020759	C	1.00	1.00
99	GATA3_141	rs57013	A	0.72	0.58

* The SNP detection reproducibility <80%

Hybridization experiments for all studied 38 individuals were repeated twice.

16% of SNPs presented only one allele in the 38 studied samples. For 14 samples (7 cases and 7 controls) the call rate for all variants was above 90%. And in one case it was below 80% due to the low quality of this particular DNA sample. For 17 SNPs the amplification step basically failed due to low quality of clinical genomic DNA samples. After exclusion of these particular 17 SNPs (indicated with an asterisk in Tab. 1) that performed poorly in hybridizations the average concordance was 93%. From the variants with high quality data, five SNPs in the genes *IL2 *(rs2069772), *TCL1B *(rs1064017), *IL11 *(rs2298885), *IL5RA *(rs2290610) and *TNFRSF1A *(rs4149570) had p-values smaller than 0.05 for the association of carrying the mutant allele with the high IgE phenotype. The homozygous genotype A for the IL5 receptor alpha (*IL5RA *Ile129 Val) was associated with a 6.8-fold risk (95% confidence interval, 1.6–29.1) of a high IgE phenotype.

## Discussion

An oligonucleotide microarray was produced using GeniomOne device to facilitate the screening of single nucleotide polymorphisms in several genes that are associated with hay-fever as a pilot project. Based on an *in silico *design, the selected set of oligonucleotides was optimized by a subsequent experimental analysis. While the *in silico *process eliminated 9% of the initially 141 SNPs that had been picked purely on the basis of a potential association with the occurrence of hay-fever, the subsequent experimental validation eliminated another 20% of these oligomers, more than twice as many. This result illustrates the importance of experimental validation of the microarray designs. Even in analyses that are based on a continuous detection of the hybridization and dissociation process (dynamic allele-specific hybridization) [18] the selection is critical, although an analysis of the association and dissociation curves of the duplexes permit a more discriminative and accurate SNP detection.

The reasons for the failing probes could be manifold [19]. Although only short fragments were hybridized, secondary structures formed either within one sample molecule or between different targets could cause inefficient binding to the array-bound probe molecules. Also, it is well known that dangling ends of the target molecules may have a profound effect on the hybridization [20]. Documentation of the effectiveness of the genotyping ability of particular sets of oligonucleotide probes is essential for a study of high accuracy. Use of flexible *in situ *synthesized oligonucleotide microarrays to such ends appears to be an efficient and attractive method for fast and cost-efficient pre-screening of candidate SNPs for an eventual high-throughput genotyping.

GeniomOne allows synthesizing 8 × 8.000 probes per array overnight and test them right after in hybridization experiments. In this way many combinations can be tested in parallel without additional cost, which allows selecting an optimal set of oligoprobes for the following experiments. This is a big advantage of GeniomOne technology.

In the analysis of the 38 DNA samples of hay-fever cases and controls, we were able to identify at least five polymorphisms in immune regulator genes that contribute to the extreme IgE phenotype and deserve further testing. For 22% of the selected SNPs, only one genotype was seen in 38 individuals. For several variants, allele-frequency differences between cases and controls exceeded 10%. These include non-synonymous variants in the IL5 receptor alpha (*IL5RA *Ile129 Val) and *TCL1B *(Gly93Arg), promoter polymorphisms in *IL2 *(-330 T/G) and *TNFRSF1A *(-609 G/T), and a polymorphism in the 3' UTR of *IL11*. *IL5RA *is a crucial factor in IL5 signalling and a contributor to the genetics of atopy in mice [21]. The extreme phenotype design of the study performed here may be an efficient alternative for the identification of disease-relevant sequence variants.

## Conclusion

Based on a platform that permits flexible *in situ *oligonucleotide synthesis, a set of optimally performing probes could be defined by a selection approach that combined computational and experimental aspects. The final design achieved by this process permitted an effective discrimination of both homo- and heterozygote polymorphisms in hay-fever patients. Allele-frequencies of more than 10% could be identified.

## Methods

### Microarray synthesis

All analysis steps, (i) *in situ *synthesis of the oligonucleotide microarray, (ii) hybridization of the labeled PCR-product mixture and (iii) detection of the signal intensities were performed with the GeniomOne device of febit biotech (Heidelberg, Germany) according to the manufacturer's instructions. The reaction carrier (DNA-processor) represents a microstructured disposable system that consists of four or eight individual arrays, respectively, which can be used individually or in any combination [8]. Controlled by a mask-free, light-controlled process, oligonucleotide probes were synthesized *in situ *in 3' to 5' direction [9]. For each selected SNP, 24 oligonucleotide probes were synthesized, 12 for either DNA strand (Fig. 1), all designed to exhibit similar hybridization characteristics. The arrays used in this study consisted of 7,448 distinct oligonucleotides (594 perfect match probes and 6,534 mismatch probes, plus 320 copies of a control oligonucleotide). A complete list can be obtained from the authors.

### PCR-amplification

For each SNP, PCR-primers were designed for the amplification of the relevant DNA-fragment using the *Primer3 *program [22]. All primers have a Tm value of 60°C. The oligonucleotides were obtained from Thermo Hybaid (Ulm, Germany). Their sequences are presented in the Table 2. The length of the PCR-products varies between 100 bp and 270 bp.

**Table 2:** Primer sequences used for PCR-amplification of the SNP-regions.

**No.**	**SNP Name**	**SNP ID**	**Forward Primer (5'-3')**	**Reverse Primer (5'-3')**
1	IL-2_1	rs2069772	CCATTCTGAAACAGGAAACCA	CTTTAAGGGGGTGGGGATAC
2	IL-2_2	rs2069763	TGCAACTCCTGTCTTGCATT	ACTTACATTAATTCCATTCAAAATCA
3	IL-10_4	rs1800894	TCCAGCCACAGAAGCTTACA	GTGCTCACCATGACCCCTAC
4	IL-10_5	rs1800871	TCCAGCCACAGAAGCTTACA	GTGCTCACCATGACCCCTAC
5	IL-10_6	rs1800872	TCCAGCCACAGAAGCTTACA	GTGCTCACCATGACCCCTAC
6	TNFA_7	rs11565	ACCACAGCAATGGGTAGGAG	CATGCCCCTCAAAACCTAT
7	TNFA_8	rs673	ACCACAGCAATGGGTAGGAG	CGTCCCCTGTATTCCATACCT
8	TNFA_9	rs1800629	GCCCCTCCCAGTTCTAGTTC	GCATCAAGGATACCCCTCA
9	TNFA_10	rs361525	GCCCCTCCCAGTTCTAGTTC	GCATCAAGGATACCCCTCA
10	IL4_11	rs2243246	GCCCCTCCCAGTTCTAGTTC	GCATCAAGGATACCCCTCA
11	IL4_12	rs2243250	AGTGAGTGGTGGGGTCCTTA	AATGCCCACTTTTTGAATGG
12	IL4_13	rs34185442	ACCCAAACTAGGCCTCACCT	GGTGGCATCTTGGAAACTGT
13	IL4_14	rs2970874	GGAAGAGAGGTGCTGATTGG	CGATTTGCAGTGACAATGTG
14	IL6_16	rs1800797	GGAAGAGAGGTGCTGATTGG	CGATTTGCAGTGACAATGTG
15	IL6_16	rs1800796	TGGCAAAAAGGAGTCACACA	CCCAAGCCTGGGATTATGAAG
16	IL6_17	rs1800795	TGGCAAAAAGGAGTCACACA	CCCAAGCCTGGGATTATGAAG
17	IL4R_18	rs1801275	GCTAGCCTCAATGACGACCT	TCATGGGAAAATCCCACATT
18	IL4R_19	rs1805011	GAAACCTGGGAGCAGATCCTC	GGCCTTGTAACCAGCCTCTC
19	IL4R_20	rs8832	AAAGGGAGCTTCTGTGCATC	TCTCCGAGCTGGTCCAG
20	IL4R_21	rs1805015	TTCCTTAGGTTGATGCTGGAG	GGTTCCATGCATACGAGGAG
21	IL4R_22	rs1805010	ACCTGACTTGCACAGAGACG	AGGGCATGTGGGTTCTACT
22	IL12p40_23	rs3124	GCCTACAGGTGACCAGCCTA	AGCCCACGGTCCAGTGTAT
23	STAT6_24	rs167769	CACAACGGAATAGACCCAAAA	ATGGCAACTTGAGAGCTGGA
24	STAT6_25	rs324015	GCACTGACTGGAAGGGAAGT	CCCTAACCTGTGCTCTTACCC
25	STAT6_26	rs703817	GTCTCAGCCCTAGGGGAATG	CTCCACCTGGCTAACAGGAA
26	STAT6_27	rs4559	CAAAAGTACAAGGGCTGA	CCCAAATTTGTGTTGTCACG
27	IFNG_28	rs2234685	GGAAGTAGGTGAGGAAGAAGCG	TGGAGCAAAGAAGGTCATCA
28	IFNG_29	rs1861493	GGAAGTAGGTGAGGAAGAAGCG	TGGAGCAAAGAAGGTCATCA
29	IFNG_30	rs2234687	TCCCATGGGTTGTGTGTTTA	GGGTCACCTGACACATTCAA
30	IFNG_31	rs2430561	TTCAGACATTCACAATTGATTTTATTC	CCCCAATGGTACAGGTTTCT
31	IFNGR2_34	rs1802585	CCCAACTCAGCCCATCTTAG	ATCTCTTCCAGGGAGCCAGT
32	IFNGR2_35	rs1059293	GGGCTGAGCAGTCAGAA	CATTTTAAGCCAGCACACCA
33	IFNGR2_36	rs9808753	CAGAGCAGGTCCTGAGTTGGGAGC	GTTTCCCACGGGTTTGATAA
34	IRF1_37	rs839	GGACTGTTCCAAAGCCAGTG	CAGAAATGTGGCAAGATCCA
35	IRF1_38	rs9282762	TTGCAAACTAAGAAAGCACACAA	ATGGGTGACACCTGGAAGTT
36	IRF2_40	rs1131553	CTCCCAAAGTGCTGGGATTA	CTGTTGTAAGGCACCGGATT
37	IL8_41	rs1175	CTTCACCATCATGATAGCATCTGT	GGAGTATGACGAAAGTTTTCTTTG
38	IL8_42	rs2227307	TGCTTTGGTAACAAACATCCTTT	GGTAACCGTCCTTCTCAATAGG
39	IL13_43	rs20541	CTTCCGTGAGGACTGAATGAGAC	CTGCAAATAATGATGCTTTCGAAGTTTCAG
40	IL13_44	rs1800925	TGACATCAACACCCAACAGG	GCAGAATGAGTGCTGCTGTGGAG
41	IL18_47	rs1946518	GGTCAGTCTTTGCTATCATTCCAG	AGCCACACGGATACCATCAT
42	IL18_48	rs1946519	GGTCAGTCTTTGCTATCATTCCAG	AGCCACACGGATACCATCAT
43	IL1B_49	rs16944	AGCCTGAACCCTGCATACC	CAATAGCCCATCCCTGTCTGT
44	IL1B_50	rs1143627	TCTCAGCCTCCTACTTCTGCTT	CAGAGAGACTCCCTTAGCACCT
45	IL1B_51	rs1799916	TCTCAGCCTCCTACTTCTGCTT	CAGAGAGACTCCCTTAGCACCT
46	IL1A_52	rs17561	CCCCCTCCAGAACTATTTTCC	ACTTTGATTGAGGGCGTCAT
47	IL1A_53	rs1800587	TAGGCTGGCCACAGGAATTA	AGCCAGAACCAGTGGCTAAG
48	IL9_56	rs1799962	CCTTCGTTAGAACACCCATGA	AGACAGGGATTCTGGTGTGA
49	TNFR1_60	rs1800692	TCCCCCTCCTGTATTCTGTG	GTGCACACGGTGTTCTGTTT
50	TNFR1_61	rs1800693	CCTGGAGTGCACGAAGTTGT	ATAGATGGATGGGTGGGATG
51	TNFRSF6_62	rs2234768	CATCCTCCTTATCCCACTTCTTT	CACCCTGTGTTTTGCATCTA
52	LTA_65	rs1800683	CACTGCCGCTTCCTCTATAA	GGTAGTCCAAAGCACGAAGC
53	LTA_66	rs1041981	CCCCCTCAACTCTGTTCTCC	GGGAGGTCAGGTGGATGTTT
54	LTA_67	rs909253	GGGTTTGGTTTTGGTTTCCT	CAGAGAAACCCCAAGGTGAG
55	IL1RN_68	rs2234676	GCCCATCTCCTCATGCTG	GCTGCTGCCCATAAAGTAG
56	IL1RN_69	rs419598	TCCTTTTCAGAATCTGGGATGT	CGTGATGCCCAGATACATTG
57	CTLA4_70	rs2384137	AACACCGCTCCCATAAAGC	CCTCCTCCATCTTCATGCTC
58	NFKBIA_72	rs1800439	CCTTGTTTTCAGCTGCCCTA	TCGTCCCCTACAAAAAGTTCA
59	IL8RB_77	rs2230054	ACATTCCAAGCCTCATGTCC	TACCAGGGCAGGCTTTCTA
60	ICAM1_78	rs1799969	CTTGAGGGCACCTACCTCTG	AGGATACAACAGGCGGTGAG
61	ICAM1_79	rs5498	CTTGAGGGCACCTACCTCTG	AGGATACAACAGGCGGTGAG
62	IL3_81	rs40401	GAGCAGTTAACCCAGCTTGTC	CACCTTGCTGCTGCACATA
63	IL3_82	rs31480	GAGCAGTTAACCCAGCTTGTC	CACCTTGCTGCTGCACATA
64	MCP1_87	rs4611511	AAAGCTGCCTCCTCAGAGTG	CACAGGGAAGGTGAAGGGTA
65	MCP1_88	rs34020694	AGAGAAAACCCGAAGCATGA	TCTTCCTAGGCCATCTCACC
66	RANTES_89	rs2107538	ATCCAGAGGACCCTCCTCAA	GGAGTGGCAGTTAGGACAGG
67	RANTES_90	rs2280788	TTCTTTTCCGTTTTGTGCAAT	CGTGCTGTCTTGATCCTCTG
68	CCR5_91	rs1799863	CTGCCTCCGCTCTACTCACT	GCCAGGTTGAGCAGGTAG
69	CCR2_94	rs1799865	AGAGGCATAGGGCAGTGAGA	GGTCCAGTTGACTGGGTGCTT
70	C5_95	rs17611	TGCAGTTTGCCCTACCTGAT	TGCTACCATTTAAGTCCTGGGTA
71	C5_96	rs17612	TTTTAGCTACAAGCCCAGCA	AATGAAGCATTCACAACACGA
72	P2X7_97	rs3751143	TTCCTGGACAACCAGAGGAG	ACCAGCTTCCTGAACAGCTC
73	IL7R_106	rs1494555	CACTATAGTTAAACCTGAGGCTCC	TCCTGGCGGTAAGCTACATC
74	PRF1_107	rs885822	CCCAGGTCAACATAGGCATCC	CGAACAGCAGGTCGTTAAT
75	TLR2_108	rs1804965	ATTCTTCTGGAGCCCATTGA	GGACTTTATCGCAGCTCTCA
76	TCL1B_109	rs1064017	ACAGTGCACTTGTGGCAG	CTGGCCATGGTCTGCTATTT
77	CCR5_110	rs1800452	TAGTCATCTTGGGGCTGGTC	TGTAGGGAGCCCAGAAGAGA
78	IL11_111	rs1126757	GGGACCACAACCTGGATTC	ATCAGAGAACACCCGACCAG
79	IL11_112	rs2298885	GGCTGTGTTCACCATAGCAA	ATCCCAAGCAAGCCTCTCTC
80	IL8RA_117	rs2234671	CATCTTTGCTGTCGTCCTCA	CCAGAATCTCAGTGGCATCC
81	IL1L1_118	rs1800930	GATGGTGCTACTGCTGTGGA	GGGCTCAGGGTAACACTG
82	CD36_119	rs1334512	CTGGCAACAAACCACACACT	TCCTACACTGCAGTCC
83	VDR_121	rs1544410	CCTCACTGCCCTTAGCTCTG	CAGGAATGTTGAGCCCAGTT
84	VDR_122	rs7975232	CTGCCGTTGAGTGTCTGTGT	ACGTCTGCAGTGTGTTGGAC
85	IL5RA_123	rs2290610	CCATGGCAATGTTTTGTCCT	CAGGTGCAGTGAAGGGAAAC
86	IL5R_124	rs2069812	CTTGCTTTTTCCTGCTGCTC	AGTCCAGGAATGGAGGCTCT
87	IL5R_125	rs2069818	TGTGGAGAAGAAAGACGGAGA	CAAAATCTTTGGCTGCAACAAACCA
88	CX3CR1_126	rs3732379	GGTGGTCATCGTGTTTTTCC	AGGCAACAATGGCTAATGC
89	CX3CR1_127	rs3732378	GGTGGTCATCGTGTTTTTCC	AGGCAACAATGGCTAATGC
90	TNFRSF1B_128	rs1061622	CTCCTGACCAAGCCTCCTC	GTCACTGGCTGGGGTAAGTG
91	TNFRSF1B_129	rs1061624	TCCTCTAGTGCCCTCCACAG	CACAGAGAGTCAGGGACTTGC
92	TNFRSF1B_130	rs3397	TCCTCTAGTGCCCTCCACAG	CACAGAGAGTCAGGGACTTGC
93	TNFRSF1A_131	rs887477	CAGCACAACTGGTCAGAACC	CCTCCTCCCAGTTCAACAAG
94	TNFRSF1A_132	rs4149570	TACAGGAACCCCAGGAGACA	TGGGTTCCAATTCAGAATGCTT
95	IL4R_135	rs1805016	GTGTCATGGCCAGGAGGAT	AGACTGGCCTCCAGTGGAAC
96	IL6_137	rs20069860	TCCCTCCACTGCAAAGGATT	CTGCAGCCACTGGTTCTGT
97	IL9_138	rs20069885	ACTTTCATCC CCACAGT	TTGCCTCTCATCCCTCTCAT
98	NKFB_139	rs1020759	TGCTTCCCTCTTGTGTTTCA	GGGGATGACCTTTAAGTGGA
99	GATA3_141	rs57013	TCCATCCATTGCACTGAGTC	CCAGAGCAGCTGGTTTAAGTG

Genomic DNA from lymphocytes was extracted using the QiaAmp Blood kit according to the manufacturer's instructions (Qiagen, Hilden, Germany). PCR-amplifications of individual loci were carried out in a Mastercycler gradient thermocycler (Eppendorf-Netheler-Hinz, Hamburg, Germany) in 25 μl of 1× Thermoprime polymerase puffer (AB Gene, Epson, UK), 200 μmol/l of each deoxynucleotide triphosphate (Qiagen), 1.5 mmol/L MgCl_2 _(AB Gene), 0.25 units Thermoprime DNA polymerase (AB Gene), 1.0 to 2.5 μmol/l of both forward and reverse primer, and 20 ng DNA. The initial annealing occurred at 94°C for 2 min, followed by 30 cycles of 94°C for 40 sec, 57°C for 40 sec and 72°C for 30 sec. Subsequently, a final extension step was performed at 72°C for 1 min.

Multiplex-PCR was performed in a total volume of 25 μl solution containing 80 ng human genomic DNA, 1.2 μmol/l of each primer, 1 mmol/l deoxynucleotide triphosphates (dNTPs), 5 mmol/l MgCl_2 _and 2 units of Thermoprime Plus DNA polymerase (AB Gene). All primer pairs had been checked *in silico *for possible primer dimers using the program "Primer Premier 5" (Premier Biosoft International, Palo Alto, USA).

DNA amplification for individuals, studied in present work, was done as described in single PCR cycling reactions. All PCR-products were checked by electrophoresis on 2% agarose gels.

### Sample processing

About 200 ng of each PCR-product were pooled and purified with the QIAquick PCR purification kit (Qiagen) according to the manufacturer's instructions. Biotin 3'-end labeling was performed as described [8]. In brief, the eluate resulting from the purification step was dried in a vacuum concentrator and the pellet was dissolved in 5 μl of water. Labeling was performed in a total volume of 10 μl with 2.5 U terminal transferase (Roche, Mannheim, Germany), 0.1 mmol/L Biotin-N^6^-ddATP (PerkinElmer, Rodgau, Germany), 2.5 nmol/l CoCl_2 _and 1× reaction buffer (Roche). After 1 h incubation at 37°C, the enzyme was inactivated at 99°C for 15 min and the mixture cooled on ice.

The final sample hybridization cocktail was made of 10 μl of this labeling reaction, 4 μl herring sperm DNA (0.1 mg/mL with 0.5 mg/ml BSA), 9 μl 2 × 2-[N-morpholino]ethanesulfonic (MES) acid buffer [54.8 mmol/l MES (free acid monohydrate), 147.7 mmol/l MES sodium salt, 1.8 mol/l NaCl, 40 mmol/l Na_2_EDTA, 0.02% (v/v) Tween20] and 6 μl water. As an internal hybridization control, 1 μl of a mixture of biotin-(50 nmol/l) and Cy5-labeled (250 nmol/l) control oligonucleotides (GCAGTGCTGCCATAACCATGAGTGA, CGCAAACTATTAACTGGCGAACTAC, GAACTGGATCTCAACAGCGGTAAGA, AAGATCAGTTGGGTGCACGAGTGGG, CGCAACAATTAATAGACTGGATGG, GCAGTGCTGCCAAAACCATGAGTGA), supplied by febit biotech, were added. The total volume of the hybridization mixture was 30 μl, which was stored frozen until use at -20°C.

### Hybridization and detection

The DNA-fragments of all SNPs of one individual person were analyzed simultaneously in a single hybridization. The biotin-labeled PCR-products in the hybridization mixture were denatured at 99°C for 5 min and quickly cooled on ice for 2 min. The probe arrays were incubated with 1 × MES solution (containing 1% BSA) at room temperature for 15 min. Then the hybridization mixture was loaded to the array. Hybridization was performed at 45°C for 4 h. Subsequently, the used sample was recovered and the array was washed with 0.5 × SSPE buffer (diluted from 6 × SSPE stock-solution consisting of 0.9 mol/l NaCl, 60 mmol/l NaH_2_PO_4 _(pH 7.4) and 6 mmol/l Na_2_EDTA) at 45°C. Staining was performed with 4 ml of 2.5 μg/ml streptavidin R-phycoerythrin conjugate (Molecular Probes, Cologne, Germany) in 6 × SSPE at room temperature for 10 minutes. All these steps were carried out automatically by the GeniomOne instrument.

### Data analysis

Image analysis was done automatically with the GeniomOne system-embedded CCD imaging system. All steps such as configuration of detection parameters, acquisition of array image, detection of feature position, calculation of signal intensity and data export to a database were performed automatically. The pattern recognition rules are digitally encoded in the analysis software, simplifying and shortening the result reading. Raw data were further processed with the integrated analysis software with the default settings.

### Statistical analysis of epidemiological data

The analysis was performed with SAS software PHREG version 9 (SAS Institute, Cary, USA). Relative risk of the elevated IgE phenotype associated with genetic variants was estimated by odds ratios (OR) and associated 95 percent confidence limits using the procedure for conditional logistic regression. The gene variants were computed as simultaneous limits of the parameters of a multinomial distribution according to Nieters et al. [23].

## Authors' contributions

JP designed and participated in the experiments and drafted the manuscript. RF contributed to the design of the experiments, conducted genotyping and imaging analysis. NB, MB and AM participated in the design and coordination of the study, contributed to the design of the experiments, conducted SNP selection and statistical analysis and in the preparation of the manuscript. JDH, AM contributed to the design of the experiments and in the preparation of the manuscript. All authors read and approved the final manuscript.
